# Data on study of hematite nanoparticles obtained from Iron(III) oxide by the Pechini method

**DOI:** 10.1016/j.dib.2019.104183

**Published:** 2019-06-25

**Authors:** Mónica Andrea Vargas, Jesús E. Diosa, Edgar Mosquera

**Affiliations:** a Departamento de Física, Universidad del Valle, A.A. 25360, Cali, Colombia; b Grupo CYTEMAC, Departamento de Física, Universidad del Cauca, Calle 5 No. 4-70, Popayán, Colombia; c Centro de Excelencia en Nuevos Materiales (CENM), Universidad del Valle, A.A. 25360, Cali, Colombia

**Keywords:** Iron(III) oxide, Hematite, Nanoparticles, Thermal and structural characterization

## Abstract

This article presents the data on α-Fe_2_O_3_ nanoparticles synthesized via Pechini method using iron(III) oxide precursor from steel industry. It is important to highlight the added value that is given to an industrial waste. The samples were characterized by thermal analysis (DTA, TG), X-ray diffraction (XRD), and Fourier transform infrared spectroscopy (FTIR). The TG showed three mass changes, whereas DTA resulted in three anomalies. X-ray diffraction pattern of the samples disclosed rhombohedral structure characteristic of the nanocrystalline α-Fe_2_O_3_ phase. The crystallite size was estimated for each thermal treatment. Fourier transform infrared spectroscopy confirms the phase purity of prepared nanoparticles. A detailed study on the local structure of the samples was carry out in the region of 800 and 400 cm^−1^, where the associated bands of Fe–O bonds are presents. The data have not been reported nor discussed for now.

**Table:** Specifications table

Subject area	Physics, Chemistry, and Materials Science
More specific subject area	Nanostructural analysis
Type of data	Figures and Table
How data was acquired	Thermal Analysis (DTA, TG); X-ray diffraction (XRD); Fourier transform infrared spectroscopy (FTIR)
Data format	Analyzed
Experimental factors	Temperature, structure, and phase were performed to determine the phase of hematite nanoparticles
Experimental features	Thermal analysis and crystal structure analysis were performed to study the synthesis of iron oxide nanoparticles. The raw material used as precursor for synthesis has been an industrial waste (oxide ferric) from steel industry.
Data source location	All data was recorded and analyzed using the equipment's from Universidad del Valle, Santiago de Cali, Colombia.
Data accessibility	Data are available within this article
Related research article	A. Lassoued, M. S. Lassoued, B. Dkhil, S. Ammar, A. Gadri. Synthesis, structural, morphological, optical and magnetic characterization of iron oxide (α-Fe_2_O_3_) nanoparticles by precipitation method: Effect of varying the nature of precursor. Physica E: Low-dimensional Systems and Nanostructures 97 (2018) 328–334 [7].

**Table:** 

**Value of the data** • Method and data will be advantageous for scale up iron oxide nanoparticles from oxide ferric obtained of the steel industry. It is important to highlight the added value that is given to an industrial waste. • The data provide structural information about hematite nanoparticles synthetized via Pechini method. • The full data and data analysis shown in this data in brief article can be used for quantitative comparison with data from other studies of similar materials exposed to different experimental conditions.

## 1 Data

**Fig. 1:**
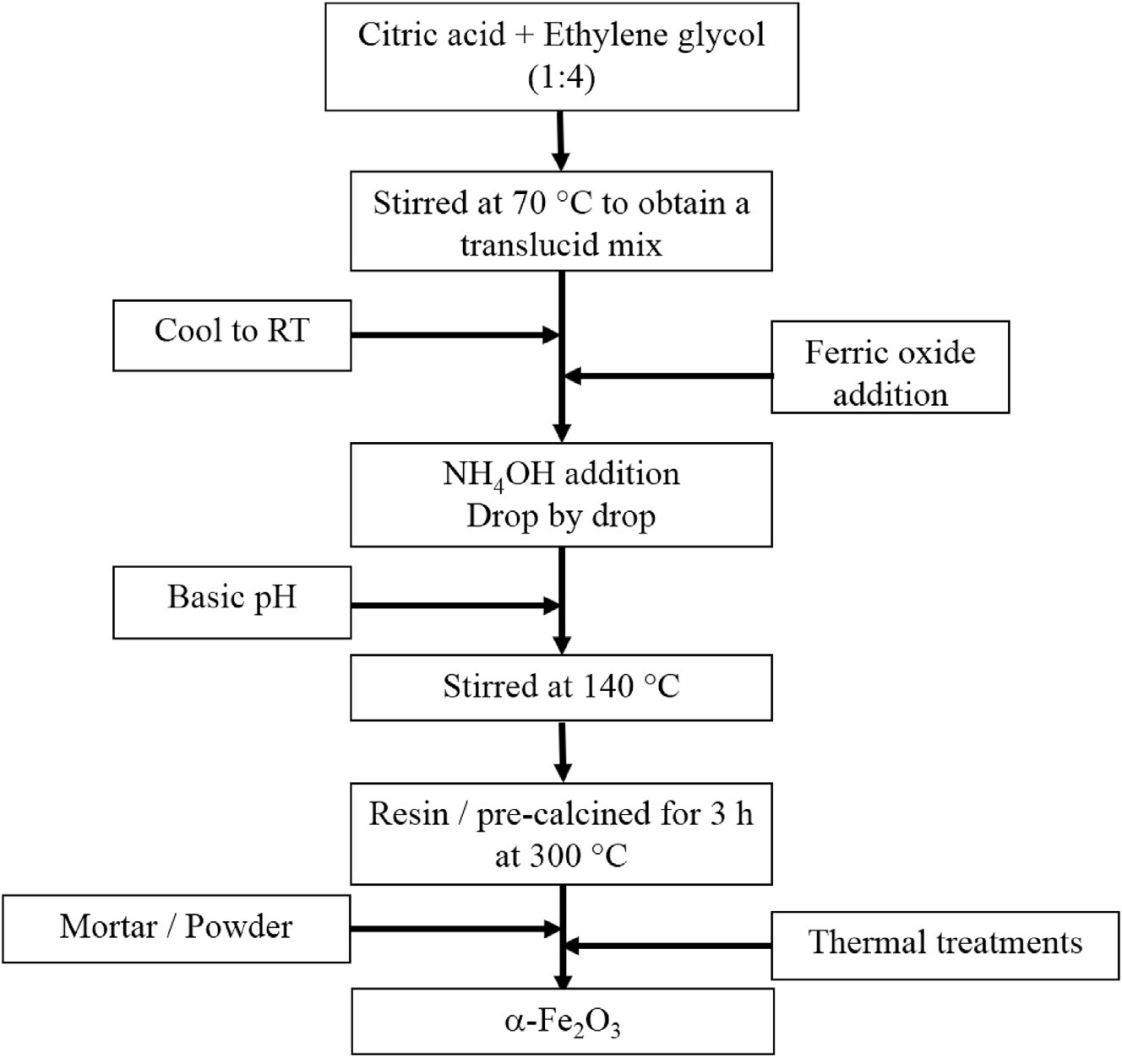
Flowchart for the synthesis of α-Fe_2_O_3_ nanoparticles by Pechini method.

**Fig. 2:**
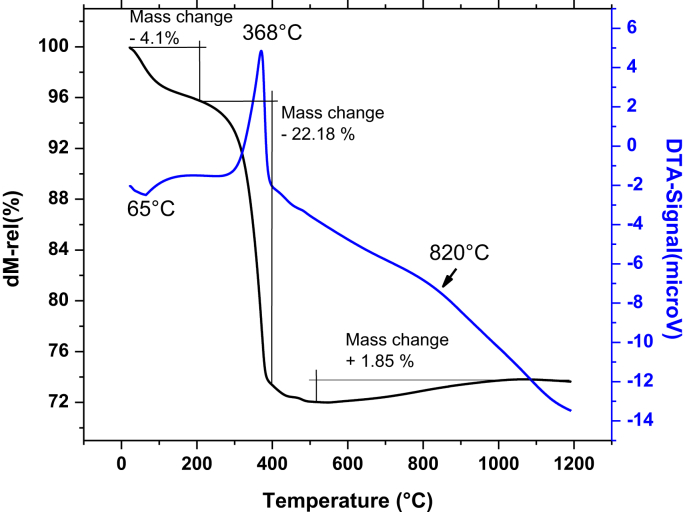
TG/DTA curves corresponding to the solid obtained of pre-calcined samples.

**Fig. 3:**
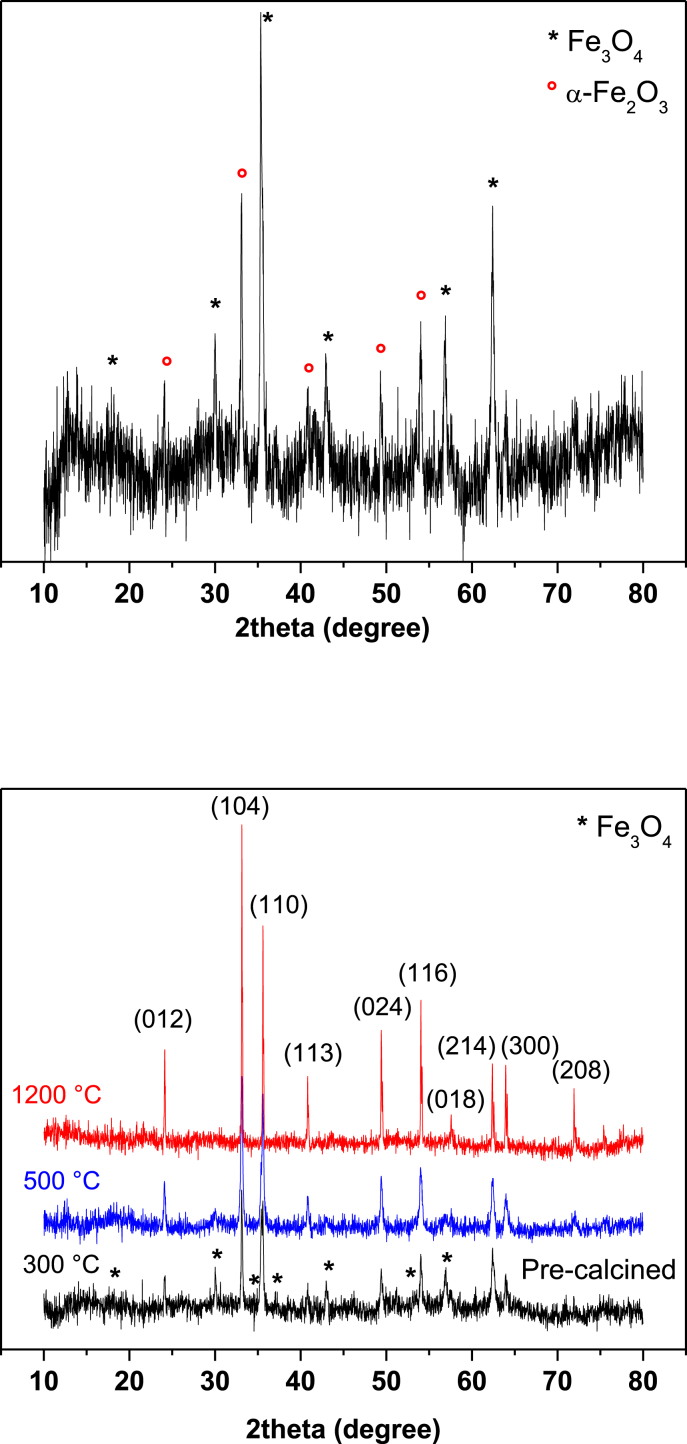
XRD patterns of the starting precursor material (up) and synthesized samples (down). The Miller index (*h k l*) of the corresponding peaks are also shown.

**Fig. 4:**
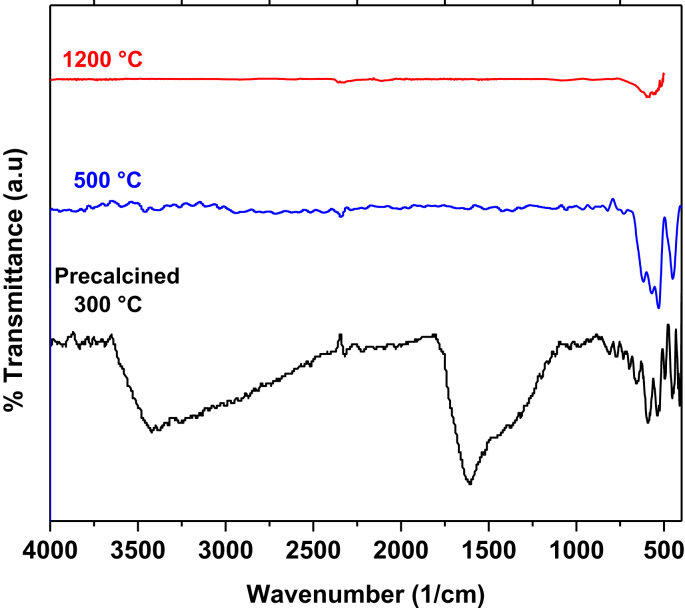
FTIR spectra of α-Fe_2_O_3_ samples heat treated at different temperatures.

**Fig. 5:**
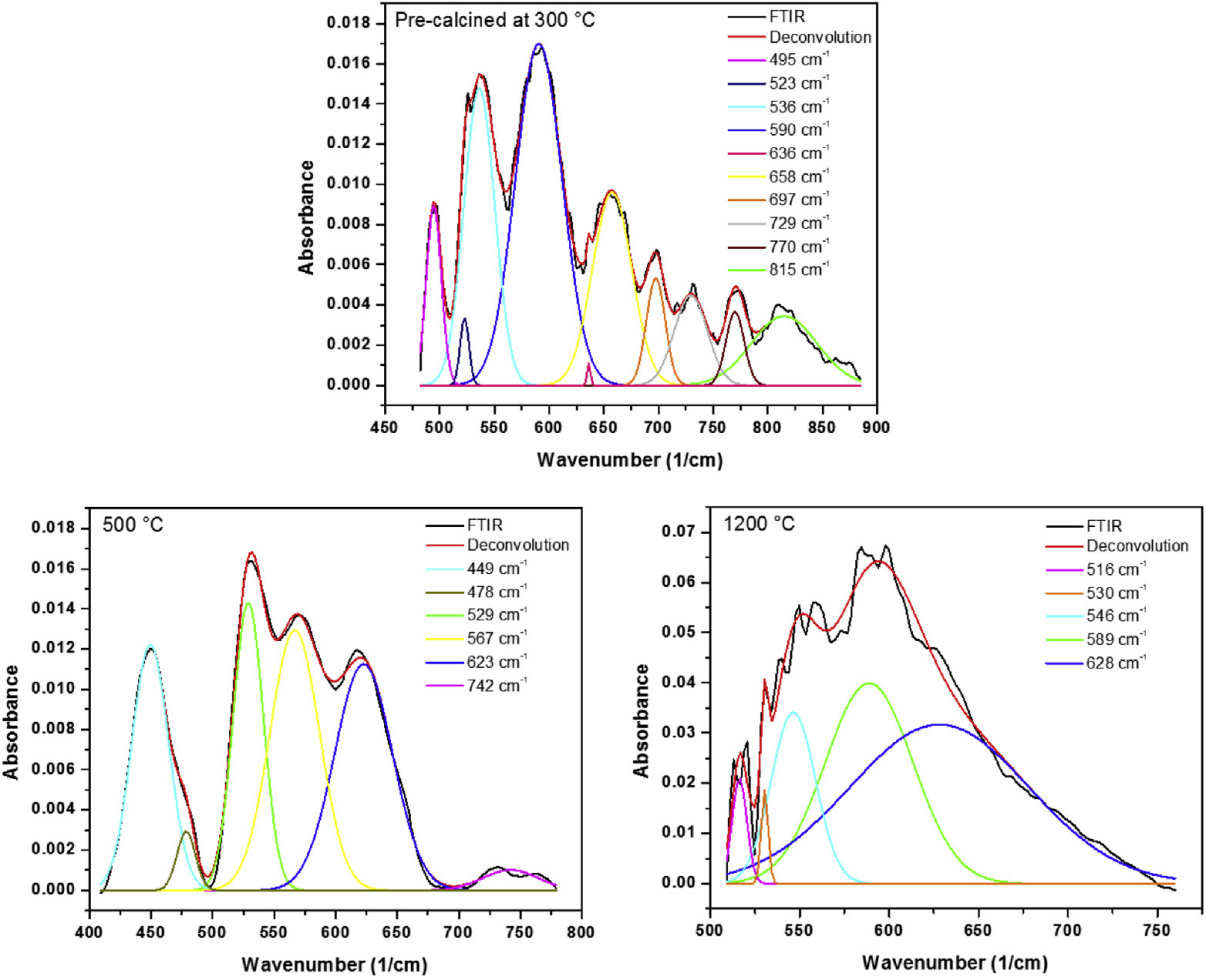
Deconvolution of the FTIR spectra, in the region between 800 and 400 cm^−1^ for the samples of α-Fe_2_O_3_ heat treated at different temperatures, 300 °C (up), 500 °C (left), and 1200 °C (right).

The data contain a description on synthesis of hematite nanoparticles via Pechini method. The synthesis procedure is shown in flowchart (Fig. 1). The precursor material was obtained from steel industry, an industrial waste. Their characterization was carried out using various analytical techniques (TG, DTA, XRD, and FTIR). Thermal analysis methods (TG/DTA) were used to study the endothermic processes (vaporization, phases transitions and chemical degradation) as well as exothermic processes (crystallization and oxidative decomposition), see Fig. 2. Additionally, structural characterization was carried out by XRD (Fig. 3 and Table 1). FTIR spectra of the samples are presented in Fig. 4. A more detailed analysis was carried out using a deconvolution of the FTIR spectra to bands at lower wavenumber than 800 cm^−1^ (Fig. 5). All corresponding Table and figures are provided with this article. This article reports the detailed data analysis from α-Fe_2_O_3_ nanoparticles.

**Table 1:** Average crystallite size obtained from XRD pattern.

Thermal treatments (T, °C)	Crystal Size (D, nm)
300	30.3
500	37.7
1200	59.3

## 2 Experimental design, materials, and methods

### 2.1 Synthesis and characterization of α-Fe_2_O_3_ nanoparticles

An oxide ferric precursor from steel industry (industrial waste) was used to synthesize hematite (α-Fe_2_O_3_) nanoparticles. The reagents were obtained from Mallinckrodt Pharmaceuticals (Ethylene glycol, P.A. 99.8%) and Merck (Citric acid, P.A. 99.5%; Ammonium hydroxide, P.A. 99%) and used as received. For characterization of the samples, the thermal analysis (TG/DTA) was performed using TA Instruments under N_2_ atmosphere, X-ray diffraction (XRD) were obtained using a PANalytical X'Pert Pro diffractometer, and an infrared spectrophotometer (Nicolet 6700 FTIR, Thermo Scientific) was used to obtain the IR spectra of the samples.

Fig. 1 shows the flowchart used for the synthesis of hematite nanoparticles via Pechini method. The precursor oxide ferric is a byproducts of the steel industry. The procedure consists in the dissolution of the precursor material in an acidic media under a basic pH under stirring at 140 °C. In this procedure, the yield was around 40% for each sample.

### 2.2 Dataset description

Thermal analysis was carry out from room temperature (RT) to 1200 °C with finality to determine the formation and decomposition phase occurring during heat treatment of synthesized samples. Fig. 2 displays the thermal analysis (TG and DTA) for the pre-calcined samples. In TG thermogram, three mass changes were observed and associated with the three anomalies showed in DTA thermogram. For TG, a first weight loss step occurred gradually between room temperature and 208 °C. The mass loss was of ∼ 4.1%, which is attributed to the elimination of water present on α-Fe_2_O_3_ nanoparticles. Additionally, the DTA analysis enabled us to find an endothermic peak at 65 °C confirming the water elimination. A second step corresponds to a mass loss of ∼ 22.18% occurring at around 208–400 °C, exothermic peak at 368 °C for DTA curve, which is associated to the volatile compounds, oxidation of the organic phase present in the samples and crystallization of the oxide. In addition, in DTA curve is observed a third peak at around 820 °C, which is associated with a little mass gain giving rise to an oxidation that is possibly associated to the loss of magnetism [1]. Based on the TG/DTA analysis, for the ceramic powders obtained by the Pechini method, it was defined that the most suitable temperature for the calcination of the material should be higher than 450 °C.

The obtained solids, precalcined at 300 °C, where thermal treated based on the results of the Fig. 2. The temperatures used for the thermal treatment was 500 °C and 1200 °C, during 4 h.

Fig. 3 shows the XRD patterns of the starting precursor material (from the steel industry) and synthesized samples. For starting precursor material and precalcined sample at 300 °C, a mixture of two phases was observed (Fig. 3(up, down)). For both samples, there are coexistence of magnetite (Fe_3_O_4_, PDF 86–1346) and hematite (α-Fe_2_O_3_, PDF 89–2810) phases. The intense peaks at 2θ values of 18.1°, 30.1°, 35.12°, 37.22°, 43.1°, 53.23°, and 57.64° corresponding to (1 1 1), (2 2 0), (3 1 1), (2 2 2), (4 0 0), (4 2 2), and (5 1 1) crystal planes are characteristic peaks of cubic structure of magnetite [2], [3].

By treating the sample thermally at 500 °C, a greater crystallization is achieved. The XRD pattern match well with the PDF no. 75–469 of the rhombohedral structure of pure hematite (α-Fe_2_O_3_) [1], [4], [5], [6], as indicated by the DTA analysis (Fig. 2). This phase is stable over a wide range of temperatures (300–1200 °C), but their magnetic property decreased with increasing of temperature (no shown). The crystallite average size was determined using the Debey-Scherrer formula [1], [4], [6], [7], and reported in Table 1.

Fig. 4 shows the FTIR spectra for α-Fe_2_O_3_ samples synthesized by the Pechini method at different temperatures. For the precalcined sample, the typical O–H bands at ∼ 3410 cm^−1^ and ∼ 1606 cm^−1^, indicate that during the resin formation, all water present in the sample was not completely eliminated, and is in agreement with thermal studies. These bands appear strongly in the spectrum at 300 °C, but their % transmission decreases as the temperature increases. In addition, this shows that the presence of the OH groups in the samples decreases as the temperature increases, disappearing in the sample treated at 500 °C. The wide band around 1370 cm^−1^ can be associated with the vibrational band of residual C–H groups [8].

Apart of the typical absorption bands around 3400 cm^−1^ and 1600 cm^−1^ due to stretching and flexion vibrational modes of hydroxyl groups, a series of absorption bands are presents in the range of 800 to 400 cm^−1^. In this region, the Fe–O vibrational bands of α-Fe_2_O_3_ at around 627, 580 and 485 cm^−1^ are presents in all samples [1], [2], [3], [4], [5]. The vibrational band at 627 cm^−1^ is due to longitudinal (Au) absorptions, while the bands at 580 cm^−1^and 485 cm^−1^ are due to the transverse absorption (Eu) of α-hematite structure. These bands are presents in the FTIR spectra of Fig. 4 [9], [10].

A detailed analysis was carried out in the region of interest (800-400 cm^−1^) using a deconvolution of the FTIR spectra, see Fig. 5. For calcined samples at 500 °C and 1200 °C, the organic species were removed after the thermal treatments. While, for precalcined sample at 300 °C, the organic phase has not been removed completely. However, the spectrum of the precalcined sample show additional vibrational bands in comparison of the sample containing α-Fe_2_O_3_ as predominant phase (Fig. 5), due to the fact that the organic phase has not been completely removed.
